# Calcium Modification During Peripheral Intervention With a Novel Intravascular Lithotripsy System: An Institutional Experience

**DOI:** 10.1016/j.jscai.2025.103708

**Published:** 2025-05-02

**Authors:** Hanad Bashir, Alan Wong, Janelle Muuse, Christian W. Schmidt, Gustavo Mendez Hirata, Christopher M. Paprzycki, Dean J. Kereiakes, John D. Corl

**Affiliations:** The Christ Hospital Heart and Vascular Institute and the Lindner Research Center, Cincinnati, Ohio

**Keywords:** calcification, intravascular lithotripsy, peripheral arterial disease

## Abstract

**Background:**

Calcium modification techniques have improved the outcomes of endovascular treatment for severely calcified stenotic lesions. In contrast to balloon angioplasty and modified balloons, intravascular lithotripsy (IVL) modifies both superficial and deep vascular calcium by delivering pulsatile sonic energy, which creates circumferential and longitudinal fractures with subsequent optimization of therapies such as stent implantation. We describe the clinical and procedural characteristics, as well as outcomes, from our initial experience with the Shockwave Javelin peripheral IVL catheter (Shockwave Medical), a novel forward IVL platform designed for difficult-to-cross calcified lesions.

**Methods:**

The first 10 patients treated with this catheter at our institution (The Christ Hospital, Cincinnati, Ohio) were included. The primary outcome was device success, defined as the ability to deliver and advance across the target lesion, pressurize, pulse, flush, and retrieve the Javelin IVL catheter. Safety outcomes included in-hospital death and procedural complication rates.

**Results:**

A total of 16 lesions in 10 patients were analyzed following IVL therapy using the Javelin device. The mean age at the time of the procedure was 74.1 ± 7.6 years. Of the 10 patients, 4 were classified as Rutherford class III, 1 as Rutherford class IV, and 5 as Rutherford class V. Additionally, 9 of 16 lesions were chronic total occlusions. All 16 lesions exhibited significant arterial calcification, with grade 3 (19%) or grade 4 (81%) calcification based on the Peripheral Arterial Calcium Scoring System (PACSS), which quantifies the severity of arterial calcification to guide treatment planning. Device success was achieved in 15/16 lesions. All patients received the maximum allowable 120 pulses with the Javelin catheter. None of the patients required a second Javelin device, but 3 of 16 lesions were treated with additional balloon-based IVL catheters. Three everolimus-eluting resorbable scaffolds were placed across 3 lesions (all below the knee). Drug-coated balloons were utilized in 5 of 16 lesions (all above the knee). Following forward IVL with the Javelin device, no arterial dissections were observed, and 3 arterial dissections occurred following subsequent balloon dilatation, none of which resulted in residual dissection after resorbable scaffold implantation. No in-hospital mortality or other postprocedural complications were noted.

**Conclusions:**

The Javelin peripheral IVL catheter appears to demonstrate high device success rates in real-world complex peripheral arterial stenoses and safely facilitates access to additional therapies. Further studies are required to better define the safety and effectiveness of Javelin IVL treatment for heavily calcified peripheral lesions.

## Introduction

Vascular calcification involves the deposition of mineralized calcium-phosphate complexes in the intimal or medial layers of vessel walls. It occurs as part of the aging process but is also associated with conditions such as hypertension, chronic kidney disease, and diabetes. Intimal calcification is the product of lipid deposition leading to macrophage invasion and activation, proliferation of smooth muscle cells, and formation of atherosclerotic plaques, which can result in obstructive vascular disease. In medial calcification, vascular smooth muscle cells differentiate into osteoblast-like cells, which then deposit mineralized calcium in the media, thereby resulting in increased arterial stiffness.^1^ Vascular calcification has also been associated with increased cardiac morbidity and mortality.^2^

In the setting of endovascular treatment for peripheral arterial disease with angioplasty and stenting, vascular calcification has been associated with increased rates of residual stenosis, dissection, perforation, and distal embolization as well as reduced antiproliferative effects of drug-coated balloons and long-term arterial patency.^3^, ^4^, ^5^, ^6^ Endovascular calcium modification tools ablate, remove, or fracture calcium to provide access to distal sites or help improve stent placement.

The Shockwave IVL catheter (Shockwave Medical) has multiple spark gap-based emitters mounted along an integrated semicompliant balloon shaft to produce vapor bubbles from the saline contrast solution inside the balloon. Cavitation of the vapor bubbles generates acoustic pressure that then radiates circumferentially and fractures superficial and deep calcium. The Shockwave IVL catheter has been shown to be safe and effective as a stand-alone or a vessel preparation strategy prior to drug-coated balloon or stent deployment for calcified peripheral artery disease.^7^, ^8^, ^9^, ^10^

Compared to high-pressure, noncompliant or modified (cutting or scoring) balloons, IVL does not require high balloon pressures (inflates to only 2-4 atm) and thus mitigates the risk of barotrauma.^11^ Atheroablative techniques are limited by wire bias in effect and predominantly modify superficial calcium, often resulting in eccentric ruts or troughs. Furthermore, atheroablation is associated with heat generation, atheroembolic particulate debris, complex vessel dissection, and perforation. Finally, IVL is able to modify both superficial and deep calcium while minimizing the risk of balloon barotauma as well as vascular complications associated with atheroablative techniques.^11^

**Figure 1:**
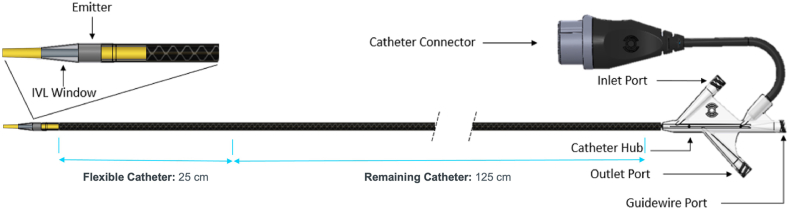
**The Shockwave Javelin Peripheral Intravascular Lithotripsy (IVL) catheter is a lithotripsy device used to treat calcified stenosis in peripheral arteries.** It generates acoustic shock waves to modify calcified plaque, facilitating artery dilation. The catheter features a forward-shifted lithotripsy emitter for localized acoustic delivery. The system includes the IVL catheter, connector cable, and generator. The catheter has a 25 cm flexible distal segment, a 150 cm working length, and is compatible with a 5F sheath (see image for components). Image courtesy of Shockwave Medical, Inc.

Currently available IVL balloon catheters have a relatively high crossing profile compared with conventional percutaneous transluminal angioplasty or percutaneous transluminal coronary angioplasty balloons, making it more challenging to navigate severe stenotic lesions or chronic total occlusions. Although their profile is comparable to older-generation devices, it remains suboptimal. The Javelin peripheral IVL catheter (Shockwave Medical) (Figure 1 and Video 1) is a novel forward IVL catheter that incorporates a single electrohydraulic sonic wave emitter behind the catheter tip to facilitate crossing of severe and/or difficult-to-cross lesions. We describe our initial institutional experience and outcomes with the Javelin peripheral IVL catheter in patients with severe stenosis and vascular calcification.

## Methods

The first 10 patients treated with this catheter at our institution (The Christ Hospital, Cincinnati, Ohio) were included. The primary outcome was device success, defined as the ability to deliver and advance across the target lesion, pressurize, pulse, flush, and retrieve the Javelin peripheral IVL catheter.^12^ Safety outcomes included in-hospital death and procedural complication rates. Continuous variables are presented as mean ± SD if normally distributed and median (IQR) if skewed. Discrete variables are reported as counts and percentages. Analyses were performed in Stata version 17.0 (StataCorp LLC).

## Results

A total of 16 lesions in 10 patients were analyzed after IVL using the Javelin device. Baseline patient and lesion characteristics are shown in Table 1. The mean age at the time of the procedure was 74.1 ± 7.6 years. Of the 10 patients, 4 were classified as Rutherford class III, 1 as Rutherford class IV, and 5 as Rutherford class V. Additionally, 9 of 16 lesions were chronic total occlusions. All 16 lesions exhibited significant arterial calcification, with grade 3 (19%) or grade 4 (81%) calcification based on the Peripheral Artery Calcium Scoring System (PACSS), which quantifies the severity of arterial calcification to guide treatment planning.

**Table 1:** Baseline clinical characteristics

Variable	N = 10
Age, y	74.1 ± 7.6
Male sex	8 (80%)
Race or ethnicity	
Black or African American	2 (20%)
White	7 (70%)
Hispanic	1 (10%)
Atrial fibrillation	3 (30%)
Paroxysmal	3/3 (100%)
Prior stroke	2 (20%)
Prior transient ischemic attack	1 (10%)
Chronic lung disease	2 (20%)
Conduction deficit	1 (10%)
Diabetes	7 (70%)
Diabetes treatment	
Diet	2/7 (29%)
Insulin	4/7 (57%)
Oral	1/7 (14%)
Rutherford clinical category	
III – Severe claudication	4 (20%)
IV – Rest pain	1 (10%)
V – Ischemic ulcers of digits	5 (40%)
Hypertension	9 (90%)
Prior myocardial infarction	2 (20%)
Peripheral artery disease	10 (100%)
Prior peripheral procedure	4 (40%)
Chronic kidney disease	3 (30%)
Stage 3	2 (20%)
End-stage renal disease	1 (10%)
Tobacco use	
Current	1 (10%)
Former	8 (80%)
Never	1 (10%)
Coronary artery disease	10 (100%)
Prior CABG	3 (30%)
No. of previous cardiac surgeries	
0	7 (70%)
1	3 (30%)
Preprocedural medications	
Aldosterone antagonist	2 (20%)
Angiotensin receptor – neprilysin inhibitor	1 (10%)
Anticoagulant	4 (40%)
Aspirin	6 (60%)
Beta blocker	6 (60%)
Loop diuretic	2 (20%)
Thiazides	3 (30%)
P2Y12 Inhibitors	4 (40%)
ACEi or ARB	7 (70%)
High-intensity statin	5 (50%)
Ezetimibe	3 (30%)
Fibrate	2 (20%)
Preprocedural laboratory testing	
Hemoglobin, g/dL	12.4 ± 2.7
Creatinine, mg/dL	1.07 (0.96-1.23)
Sodium, mmol/L	138.9 ± 4.1
Platelet count per L	238,600 ± 102,244
LDL cholesterol, mg/dL	62.5 (54-136)

Values are mean ± SD, n (%), or median (IQR).

ACEi, angiotensin converting enzyme inhibitors; ARB, angiotensin II receptor blocker; CABG, coronary artery bypass grafting; LDL, low-density lipoprotein.

### Procedure characteristics

Nine of 16 lesions were chronic total occlusions, and 10 of 16 lesions treated were below the knee. All patients received the maximum allowable 120 pulses with the Javelin catheter. None of the patients required a second Javelin device, but 3 of 16 lesions were treated with additional balloon-based IVL. Three Everolimus-eluting resorbable scaffolds were placed across 3 lesions (all below the knee). Drug-coated balloons were utilized in 5 of 16 lesions (all above the knee) (Table 2).

**Table 2:** Procedural characteristics.

Characteristics	N = 16 lesions
Preprocedural CTO	9 (56%)
PACSS	
Grade 3	3 (19%)
Grade 4	13 (81%)
Vessel-specific anatomy	
Above the knee	6 (38%)
Below the knee	10 (63%)
Maximum pressure used in Javelin, atm	4 (4-6)
Mean No. of Javelin pulses delivered	120
Postdilation performed	16 (100%)
No. of balloon-based IVL used after Javelin	3 (19%)
Drug-coated balloon used for lesions that underwent Javelin	5 (38%)
Total no. of stents implanted to lesions that underwent Javelin	3 (19%)
Device success	15 (94%)
Moderate sedation	16 (100)
Procedure time, min	143 (123-156)
Dose area product, Gy × cm^2^	38.4 ± 28.7
Cumulative air kerma, mGy	185.9 ± 140.0
Fluoroscopy time, min	23.8 ± 7.5
Contrast volume, mL	81.1 ± 16.4
Length of stay, d	0.5 (0-4)

Values are n (%), median (IQR), or mean ± SD.

CTO, chronic total occlusion; IVL, intravascular lithotripsy; PACSS, Peripheral Artery Calcium Scoring System.

### Procedure outcomes and complications

Device success was achieved in 15 of 16 lesions treated. The unsuccessful lesion was severely calcified and uncrossable with any wire, device, or technique. After IVL with the Javelin device, no arterial dissections were observed. However, 3 arterial dissections occurred after subsequent balloon dilatation, none of which resulted in residual dissection after destination therapy. No in-hospital mortality or other postprocedural complications were noted (Table 3).

**Table 3:** Procedural complications

Intraprocedural or postprocedural complications	N = 10
Arterial dissection post-dilation	3/16 (19%)
Arterial dissection post-Javelin	0
Arterial dissection final images	0
Arterial perforation	0
Bleeding – access site	1 (10%)
Blood transfusion	0
Abrupt vessel closure	0
Distal embolization	0
Above the ankle amputation	0
Thrombus	0
No reflow	0
Arterial spasm	0
Death	0
Discharge medications	
Aldosterone antagonist	2 (20%)
Angiotensin receptor-neprilysin inhibitor	1 (10%)
Anticoagulant	4 (40%)
Aspirin	8 (80%)
Beta blocker	6 (60%)
Loop diuretic	4 (40%)
Thiazides	2 (20%)
P2Y12 inhibitors	9 (90%)
ACEi or ARB	7 (70%)
High-intensity statin	5 (50%)
Ezetimibe	4 (40%)
Fibrate	2 (20%)

Values are n (%).

ACEi, angiotensin converting enzyme inhibitors; ARB, angiotensin II receptor blocker.

## Discussion

This single-center study of consecutive patients treated with the Javelin peripheral IVL catheter was performed to evaluate the procedural characteristics as well as angiographic and clinical outcomes from our initial experience with this novel forward IVL platform designed to facilitate treatment of difficult-to-cross, severely calcified lesions. The main findings of this study are (1) the Javelin peripheral IVL catheter demonstrated a high rate of device success; and (2) this device appears to safely facilitate access for additional therapies with low complication rates.

Severely calcified peripheral vessels portend risk and challenges during peripheral intervention, leading to poor procedural success rates and patient outcomes. To address these challenges, multiple calcium modification techniques have been created to facilitate treatment of severely calcified lesions, with the goal of improving procedural and clinical outcomes.^11^

Intravascular lithotripsy has emerged as a novel therapy for the treatment of severe vascular calcification. IVL uses acoustic pressure waves delivered through a balloon angioplasty catheter to circumferentially and longitudinally fracture vascular calcium to facilitate revascularization. The safety and effectiveness of IVL in modifying heavily calcified lesions have been described in multiple studies (Disrupt PAD I, II, III, and Disrupt BTK).^7^^,^^10^^,^^13^, ^14^, ^15^ However, IVL balloon catheters are relatively high-profile and may face challenges in crossing severely stenotic lesions or chronic total occlusions.

The Javelin peripheral IVL catheter is a non–balloon-based catheter with a forward-shifted, single electrohydraulic emitter proximal to the catheter tip that is delivered over a 0.014 inch standard guide wire positioned across the target stenosis. This allows localized delivery of acoustic pressure waves closer to difficult-to-cross lesions compared with balloon-based IVL platforms. Javelin was designed to be used in severely calcified, severely stenosed vessels in which the use of other devices may be limited or may expose the patient to an increased risk of complications.

The Javelin IVL catheter has been shown to have comparable effectiveness and safety outcomes to balloon-based IVL. The FORWARD PAD IDE study and Mini S Feasibility study included 103 treated lesions; in these studies, the mean diameter stenosis was 82.9 ± 16.7% with a mean lesion length of 76.9 ± 59.4 mm. Furthermore, 38% of lesions were chronic total occlusions, 82.5% were severely calcified, and 42.7% were located below the knee. The Javelin IVL catheter was able to achieve complete crossing in 93% of lesions, with a technical success rate of 99%, which exceeded the prespecified performance goal of 85% (*P* < .0001), and it reduced baseline stenosis by 23 ± 9.1%. The primary safety end point of major adverse events at 30 days was 1.1%, which was better than the prespecified performance goal of 11.2% (*P* = .0012).^12^ Regarding balloon-based IVL, a meta-analysis of 9 studies, which included 681 patients treated who underwent balloon-based IVL, showed a statistically significant decrease in the mean diameter stenosis percentage after IVL. In addition, the overall pooled event rate for flow-limiting or type D, E, or F dissection was 1.25%, and there was no procedure-related or device-related death, limb loss, or major amputation reported in the studies.^16^

### Limitations

The findings from this first commercial-use study should be considered in the context of several important limitations. First, given the single-center, retrospective nature of our observations and the small sample size, our findings may be considered definitive with respect to the safety and effectiveness of this novel IVL catheter. Second, longer-term follow-up data are not available. Larger studies with longer-term follow-up are required to definitively establish the safety and effectiveness of the Javelin catheter for treatment of a broad spectrum of severely calcified and stenosed vessels.

## Conclusion

The Javelin peripheral IVL catheter demonstrated a high rate of device success in real-world clinical practice involving anatomically complex cases and appears to safely facilitate access to additional therapies. These results are similar to those observed in the FORWARD PAD IDE study. Further studies confirming Javelin’s safety and efficacy in real-world settings with diverse patient and lesion populations will strengthen its position as a novel treatment option for the most complex forms of calcified peripheral artery disease. There are important findings to this study (Central Illustration).

**Central Illustration:**
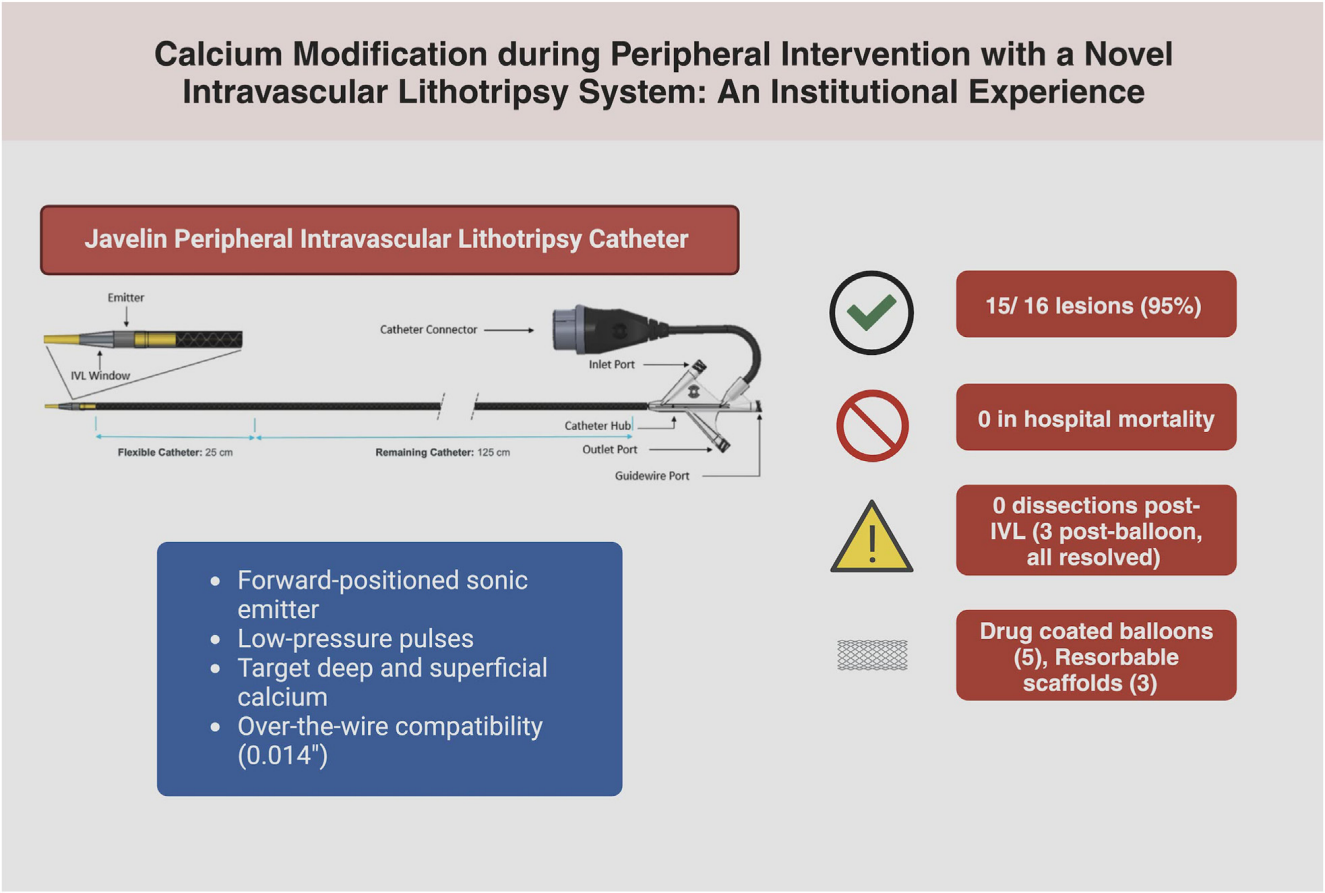
**Javelin IVL catheter achieved 95% lesion success with no in-hospital mortality or post-IVL dissections in this institutional experience.**

## Supplementary material

Video 1 The Javelin forward intravascular lithotripsy device crossing a calcified popliteal artery.
